# The new oral anti-coagulants and the phase 3 clinical trials - a systematic review of the literature

**DOI:** 10.1186/1477-9560-11-18

**Published:** 2013-09-03

**Authors:** Faryal Tahir, Haris Riaz, Talha Riaz, Maaz B Badshah, Irbaz B Riaz, Ameer Hamza, Hafsa Mohiuddin

**Affiliations:** 1 Dow Medical College, Dow University of Health Sciences, Baba-e-Urdu Road, Karachi, Pakistan; 2 Department of Medicine, Indiana University, Indianapolis, Indiana, USA; 3 Department of Medicine, University of Arizona, Arizona, USA; 4 Quaid-e-Azam Medical College, Bahawalpur, Pakistan

**Keywords:** Vitamin K antagonists, Oral anticoagulants, Apixaban, Rivaroxaban, Dabigatran, Orthopedic surgery, Knee replacement, Hip replacement, Acute coronary syndrome, Atrial fibrillation, Venous thromboembolism, Critically ill patients, Systematic review

## Abstract

**Background:**

Anticoagulation with vitamin K antagonists such as warfarin has historically been used for the long term management of patients with thromboembolic disease. However, these agents have a slow onset of action which requires bridging therapy with heparin and its analogues, which are available only in parenteral route. To overcome these limitations, new oral anticoagulants such as factor Xa inhibitors and direct thrombin inhibitors have been developed. The aim of this article is to systematically review the phase 3 clinical trials of new oral anticoagulants in common medical conditions.

**Methods:**

We searched PubMed (Medline) from January 2007 to February 2013 using “Oral anticoagulants”, “New oral anticoagulants”, “Randomized controlled trial”, “Novel anticoagulants”, “Apixaban”, “Rivaroxaban”, “Edoxaban”, “Dabigatran etexilate”, “Dabigatran” and a combination of the above terms. The available evidence from the phase 3 RCTs was summarized on the basis of individual drug and the medical conditions categorized into “atrial fibrillation”, “acute coronary syndrome”, “orthopedic surgery”, “venous thromboembolism” and “medically ill patients”.

**Results:**

Apixaban, rivaroxaban and dabigatran have been found to be either non-inferior or superior to enoxaparin in prophylaxis of venous thromboembolism in knee and hip replacement with similar bleeding risk, superior to warfarin for stroke prevention in atrial fibrillation with significant reduction in the risk of major bleeding, non-inferior to aspirin for reducing cardiovascular death and stroke in acute coronary syndrome with significant increase in the risk of major bleed. Rivaroxaban and dabigatran are also superior to the conventional agents in the management of symptomatic venous thromboembolism. However, compared to enoxaparin, apixaban and rivaroxaban use lead to significantly increased bleeding risk in medically ill patients. Additional studies evaluating the specific reversal agents of these new drugs for the management of life-threatening bleeding or other adverse effects are necessary.

**Conclusion:**

Considering their pharmacological properties, their efficacy and bleeding complications, the new oral agents offer a net favourable clinical profile in orthopedic surgery, atrial fibrillation, acute coronary syndrome and increase the risk of bleeding in critically ill patients. Further studies are necessary to determine the long term safety and to identify the specific reversal agents of these new drugs.

## Introduction

Thromboembolic (TE) disease is a common cause of morbidity and mortality. Vitamin K antagonists (VKAs) such as warfarin are traditionally used for the prolonged management of thromboembolic disease. However, given the slow onset of action of these oral medications, a bridging therapy with either unfractioned or low molecular weight heparin (LMWH) is routinely used. LMWH is associated with a reduced rate of adverse effects and have replaced the use of heparin for several indications.

Since heparin and LMWH are available only for parenteral use, continuous attempts have been made to develop the oral alternatives of these medications. The present article discusses the new oral anticoagulants. We have summarized the findings of phase 3 trials on the new oral anticoagulants. A brief overview of the pharmacological properties of these agents is also presented.

### What is the necessity of new oral anticoagulants?

**Figure 1:**
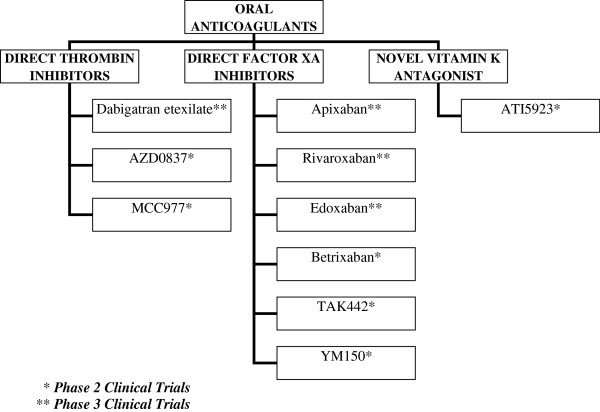
**Classification of oral anticoagulants in development.** * Phase 2 Clinical Trials. ** Phase 3 Clinical Trials.

Given the need for subcutaneous route of administration, long term use of LMWH poses a problem. The new oral anticoagulants specifically target either thrombin or factor Xa (Figure 1), whereas warfarin inhibits synthesis of all vitamin K-dependant clotting factors. Moreover, in contrast to warfarin, the new oral anticoagulants produce a predictable anticoagulant effect that does not require frequent laboratory monitoring in order to adjust therapy. In addition, along with a rapid onset and offset of action, these agents have low potential for dietary interactions. As a result, the new oral agents are more convenient to administer than warfarin and therefore may limit the long term use of warfarin.

## Literature search criteria

**Figure 2:**
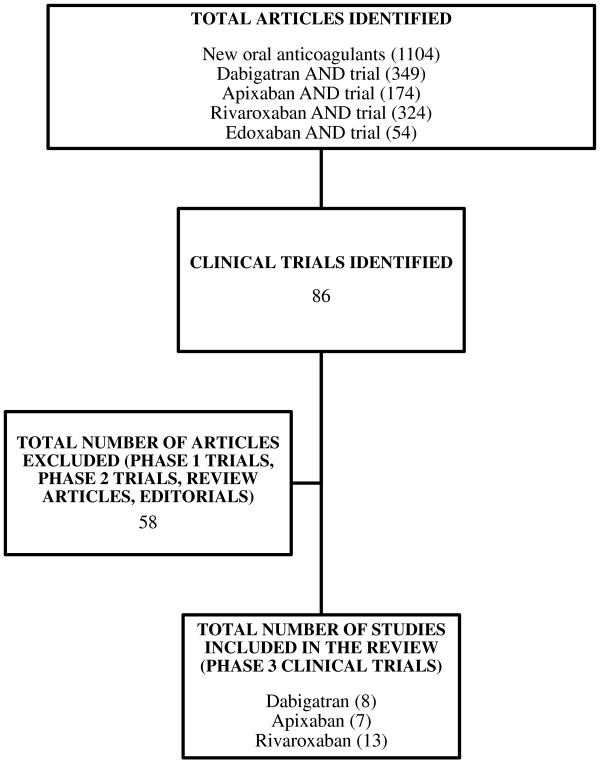
Inclusion of phase 3 clinical trials for review.

A review of the literature was carried out from the PubMed database using the search terms “Oral anticoagulants”, “New oral anticoagulants”, “Randomized controlled trial”, “Novel anticoagulants”, “Apixaban”, “Rivaroxaban”, “Edoxaban”, “Dabigatran etexilate” and “Dabigatran” and a combination of the above terms. The articles were then manually examined to exclude the duplicate entries. Search was limited from January 2007 to February 2013. All the retrieved articles in English language were further searched to include the phase 3 randomized controlled trials. Observational studies, phase 1 and phase 2 trials were excluded. We also excluded personal opinions, editorials, correspondences and perspective articles. Full text versions of the included articles were downloaded and evaluated by the authors to compile a narrative review on the field (Figure 2). The available evidence from the phase 3 RCTs was summarized on the basis of individual drug and the medical conditions categorized into “atrial fibrillation”, “acute coronary syndrome”, “orthopedic surgery”, “venous thromboembolism” and “medically ill patients”. Relevant details of the pharmacokinetic properties of the drugs were added to complete the drug profile independent of the above mentioned literature search.

## Apixaban

Apixaban, a highly selective and direct acting, reversible factor Xa inhibitor, has rapid absorption and a half life of approximately 12 hours [1-3]. The absorption of this drug is not affected by food intake and it is eliminated primarily through fecal (75%) but also through renal (25%) pathway [4]. The drug undergoes metabolism via cytochrome isoform CYP3A4, therefore, concomitant therapy with potent CYP3A4 inhibitors, such as azole antifungals, macrolide antibiotics and protease inhibitors is contraindicated in patients receiving apixaban. In phase 2 clinical trials, apixaban has been shown to provide effective and predictable anticoagulation over the doses of 5, 10 or 20 mg/day in the prevention and treatment of venous thromboembolism [5,6]. Another phase 2 trial demonstrated the efficacy of two doses of apixaban (2.5 mg and 5 mg BID) compared with adjusted-dose warfarin in patients with nonvalvular atrial fibrillation [7].

### Medically ill patients

The Apixaban Dosing to Optimize Protection from Thrombosis (ADOPT) [8] trial randomized 6528 patients with congestive heart failure, respiratory failure or other medical disorder with atleast one risk factor for VTE to receive either apixaban 2.5 mg once daily for 30 days or subcutaneous enoxaparin 40 mg once daily for 6 to 14 days. However, the extended course of thromboprophylaxis resulted in significantly increased (p < 0.05) bleeding events (0.47% patients in the apixaban group vs. 0.19% in the enoxaparin group; relative risk, 2.58; 95% CI, 1.02 to 7.24).

### Orthopedic surgery

Apixaban for the Prevention of Thrombosis-related Events (ADVANCE) 1 [9] assigned 1599patients to receive 2.5 mg twice daily apixaban 12 to 24 hours following knee replacement surgery while 1596 patients received enoxaparin 30 mg subcutaneously twice daily. The rate of primary efficacy outcome was similar in the 2 groups (9.0% in the apixaban group compared to 8.8% in the enoxaparin group). However, the apixaban group was associated with a significantly (p < 0.05) reduced incidence of major and clinically relevant non-major bleeding episodes (2.9% vs. 4.3%).

The ADVANCE 2 [10] recruited 3057 knee replacement patients to receive the same apixaban dose or enoxaparin 40 mg daily. This study reported a significantly reduced incidence of total VTE in the apixaban group (15.1%) compared with enoxaparin (24.4%) (p < 0.0001; absolute risk reduction 9.3% [5.8-12.7]) with a non-significantly (p = 0.09) reduced risk of bleeding (4% vs. 5%).

The Apixaban Dosed Orally Versus Anticoagulation with Injectable Enoxaparin to Prevent Venous Thromboembolism 3 (ADVANCE-3) trial [11] compared 5407 patients undergoing hip arthroplasty to receive either 2.5 mg twice daily apixaban or 40 mg once daily enoxaparin for 5 weeks post-surgery. The primary efficacy outcome occurred in 1.4% patients in the apixaban group compared with 3.9% in the enoxaparin group (relative risk with apixaban, 0.36; 95% CI, 0.22 to 0.54; p < 0.001 for both noninferiority and superiority; absolute risk reduction, 2.5 percentage points; 95% CI, 1.5 to 3.5). The incidence of major and clinically relevant non-major bleeding events was similar in the two groups (4.8% in the apixaban group vs. 5.0% in the enoxaparin group).

### Atrial fibrillation

Apixaban for Reduction in Stroke and Other Thromboembolic Events in Atrial Fibrillation (ARISTOTLE) trial [12] was a comparison between apixaban (5 mg twice daily) and warfarin (titrated to an INR of 2.0 to 3.0). The study enrolled a total of 12,801 patients with atrial fibrillation and at least one risk factor for stroke for a median duration of 1.8 years. 1.27% patients in the apixaban group while 1.6% in the warfarin group experienced primary outcome (hazard ratio with apixaban, 0.79; 95% CI, 0.66 to 0.95; p < 0.001 for noninferiority; p = 0.01 for superiority). The incidence of hemorrhage in the apixaban group was 0.24% compared to 0.47% in the warfarin group (hazard ratio, 0.51; 95% CI, 0.35 to 0.75; p < 0.001). The rates of death in the two groups were 3.52% and 3.94%, respectively (p = 0.047).

The AVERROES [13] (Apixaban Versus Acetylsalicylic Acid [ASA] to Prevent Stroke in Atrial Fibrillation Patients Who Have Failed or Are Unsuitable for Vitamin K Antagonist Treatment) evaluated apixaban 5 mg twice daily compared with aspirin (81 to 324 mg per day) for a median duration of 1.1 years in 5599 patients with atrial fibrillation in which vitamin K antagonist therapy was unsuitable. The study was terminated earlier because the apixaban group had significantly reduced primary outcome events (1.6% per year compared to 3.7% in the aspirin group) (hazard ratio with apixaban, 0.45; 95% CI, 0.32 to 0.62; p < 0.001). Incidence of major bleeding was 1.4% per year in the apixaban group whereas 1.2% in the aspirin group.

### Acute coronary syndrome

To compare apixaban versus standard antiplatelet therapy in patients with acute coronary syndrome (ACS) with at least two risk factors for recurrent ischemia, the Apixaban for Prevention of Acute Ischemic Events 2 (APPRAISE-2) [14] trial was conducted. It included 7392 patients who were taking aspirin or aspirin plus clopidogrel after an ACS. These patients were randomly assigned in a 1:1 ratio to receive apixaban 5 mg twice daily or matching placebo. However, this study was terminated prematurely due to significantly increased bleeding incidence (1.3%) in the apixaban group compared to 0.5% in the antiplatelet group (hazard ratio with apixaban, 2.59; 95% CI, 1.50 to 4.46; p = 0.001). The primary outcome of cardiovascular death, myocardial infarction (MI) or ischemic stroke occurred in the 7.5% patients assigned in the apixaban group compared to 7.9% in the placebo group (p = 0.51).

## Rivaroxaban

Rivaroxaban is another oral, direct acting, reversible factor Xa inhibitor with a rapid onset of action and dose-proportional pharmacokinetics and pharmacodynamics [15,16]. It has a half-life of up to 12 hours in elderly patients, and up to 9 hours at steady state in young healthy subjects [2]. Food does not affect its absorption from the gastrointestinal tract. It has a dual mode of elimination, since one-third of the active drug is excreted unchanged renally while two-thirds of the drug undergoes hepatic metabolism. Like apixaban, it interacts with potent inhibitors of cytochrome P450 enzyme CYP3A4; however, unlike apixaban, it also interacts with potent inhibitors of P-glycoprotein, therefore drugs like quinidine are contraindicated in rivaroxaban-treated patients [4]. It should be used cautiously in patients with severely impaired renal function. It has no effect on the QTc interval [17].

### Medically ill patients

In the MAGELLAN (Multicenter, rAndomized, parallel Group Efficacy and safety study for the prevention of VTE in hospitalized acutely iLL medical patients comparing rivaroxabAN with enoxaparin) [18] study, a total of 8101 patients aged ≥ 40 years with acute medical illness were randomized to receive 40 mg subcutaneous enoxaparin once a day for 10 ± 4 days and oral placebo for 35 ± 4 days or subcutaneous placebo for 10 ± 4 days and 10 mg oral rivaroxaban once daily for 35 ± 4 days for VTE prophylaxis. A primary efficacy outcome (composite of asymptomatic proximal or symptomatic venous thromboembolism) occurred in 2.7% of the patients in each group at day 10 (relative risk with rivaroxaban, 0.97; 95% CI, 0.71 to 1.31; p = 0.003 for noninferiority) and in 4.4% of patients receiving rivaroxaban and in 5.7% of patients receiving enoxaparin followed by placebo at day 35 (relative risk, 0.77; 95% CI, 0.62 to 0.96; p = 0.02 for superiority). Rivaroxaban demonstrated more clinically relevant bleeding as compared to enoxaparin at day 10 (2.8% vs. 1.2%; p < 0.001) and at day 35 (4.1% vs. 1.7%; p < 0.001).

### Orthopedic surgery

The Regulation of Coagulation in Orthopedic Surgery to Prevent Deep Venous Thrombosis and Pulmonary Embolism 1 (RECORD 1) [19] trial compared the efficacy and safety of 10 mg oral rivaroxaban once daily initiated postoperatively to 40 mg subcutaneous enoxaparin once daily initiated preoperatively in the prevention of VTE in 4541 patients undergoing total hip arthroplasty. The primary efficacy outcome occurred in 1.1% patients in the rivaroxaban group compared with 3.7% in the enoxaparin group (absolute risk reduction, 2.6%; 95% CI, 1.5 to 3.7; p < 0.001). Major bleeding events occurred in 0.3% patients in the rivaroxaban group and in 0.1% patients in the enoxaparin group (p = 0.18).

The RECORD 2 [20] trial compared extended duration rivaroxaban (31–39 days) to short-term enoxaparin (10–14 days) in 2509 patients undergoing total hip arthroplasty. Again, rivaroxaban was associated with a lower incidence of VTE and all-cause mortality (2% vs. 9.3%; absolute risk reduction 7.3%, 95% CI, 5.2-9.4; p < 0.0001). The incidence of major bleeding events was much the same in both groups (6.6% in the rivaroxaban group vs. 5.5% in the enoxaparin group; p = 0.25).

The RECORD 3 [21] study recruited 2531 knee replacement patients to receive the same dose of oral rivaroxaban or subcutaneous enoxaparin once daily. Rivaroxaban was superior to enoxaparin in preventing VTE and all-cause mortality 13–17 days after surgery (9.6% vs. 18.9%; absolute risk reduction, 9.2%; 95% CI, 5.9 to 12.4; p < 0.001). Major bleeding events were similar (0.6% rivaroxaban vs. 0.5% enoxaparin).

In the RECORD 4 [22] trial, 3148 patients undergoing knee arthroplasty were randomized to receive the same dose of rivaroxaban once daily and the North American enoxaparin regimen (2 × 30 mg/d), compared to the European regimen (1 × 40 mg/d) used in the previous RECORD trials. This trial also demonstrated that rivaroxaban had significantly reduced primary outcome events (6.9% vs. 10.1%; absolute risk reduction 3.19%, 95% CI 0.71-5.67; p = 0.0118) and major bleeding events were comparable (0.7% rivaroxaban vs. 0.3% enoxaparin; p = 0.1096). These phase 3 trials showed comparable safety and superior efficacy of rivaroxaban compared with enoxaparin for thromboprophylaxis after hip or knee arthroplasty.

### Atrial fibrillation

The Rivaroxaban Once Daily Oral Direct Factor Xa Inhibition Compared with Vitamin K Antagonism for Prevention of Stroke and Embolism Trial in Atrial Fibrillation (ROCKET AF) [23,24] was conducted in 14,264 patients of non-valvular atrial fibrillation. The trial suggested that at a dose of 20 mg (15 mg in patients with creatinine clearance of 30 to 49 ml/min), the rivaroxaban group was associated with significantly reduced incidence of stroke or systemic embolism compared with the warfarin group (hazard ratio in the rivaroxaban group, 0.79; 95% CI, 0.66 to 0.96; p < 0.001 for noninferiority). Similarly, the incidence of fatal bleeding (0.2% vs. 0.5%, p = 0.003) and intracranial hemorrhage (0.5% vs. 0.7%, p = 0.02) was significantly reduced in the rivaroxaban group compared to warfarin.

### Acute coronary syndrome

On the basis of Anti-Xa Therapy to Lower Cardiovascular Events in Addition to Standard Therapy in Subjects with Acute Coronary Syndrome–Thrombolysis in Myocardial Infarction 46 (ATLAS ACS–TIMI 46), a phase 2 trial of rivaroxaban versus placebo to determine the dose of rivaroxaban in ACS patients [25], a phase 3 trial designated ATLAS ACS 2–TIMI 51 was conducted [26]. In this trial, 15, 526 patients were randomized to receive either 2.5 mg or 5 mg placebo twice daily for a mean duration of 13 months. The trial suggested that rivaroxaban significantly (p = 0.008) reduced the primary end point (death from cardiovascular causes, MI or stroke). However, rivaroxaban increased the risk of non-fatal major bleeding (2.1% vs. 0.6%, p < 0.001) and intracranial hemorrhage (0.6% vs. 0.2%, p = 0.009).

## Symptomatic venous thromboembolism

After the initial feasibility studies [27,28] the EINSTEIN clinical trials program was initiated to test the efficacy of rivaroxaban as a single agent for the management of symptomatic VTE. The program comprised of 3 trials.

The results of the Acute DVT Study [29] showed that rivaroxaban (15 mg twice daily for 3 weeks, followed by 20 mg once daily) had non-inferior efficacy compared with enoxaparin plus vitamin K antagonist therapy in terms of the primary outcome (recurrent venous thromboembolism) (2.1% events in the rivaroxaban group versus 3.0% in the control group; p < 0.001).

The Continued Treatment Study (EINSTEIN-Extension) [29] showed that rivaroxaban had superior efficacy than the control group (1.3% versus 7.1% events; p = 0.001).

In the Einstein-PE study [30], rivaroxaban was noninferior to the standard therapy with enoxaparin and vitamin K antagonist for the primary efficacy outcome in patients with acute symptomatic pulmonary embolism with or without deep-vein thrombosis (2.1% events in the rivaroxaban group versus 1.8% in the control group; p = 0.003).

## Edoxaban

Edoxaban is a rapidly absorbed active drug with a half life of 9–11 hours [4,31]. An RCT has demonstrated that the effect of food on the pharmacokinetic properties of the drug is clinically insignificant [32]. The drug is eliminated both via kidney (one third) and in feces (two third). A double blind Phase 2 RCT of Edoxaban versus subcutaneous daltaperin has demonstrated significantly reduced incidence (p < 0.001) of venous thromoembolism after elective total hip replacement at both low (15 and 30 mg) and higher (60 and 90 mg) doses of edoxaban [33]. Similarly, the drug significantly reduced the incidence of VTE in patients of total knee arthroplasty when compared with placebo while the rates of bleeding were similar in both groups [34]. Another phase 2 study showed that the safety profiles of 30 and 60 mg qd doses of the drug were similar when compared with warfarin [35]. The ENGAGE-AF-TIMI48 is a phase 3 noninferiority design megatrial that is comparing edoxaban (30 mg or 60 mg once daily) or warfarin (titrated to an INR of 2.0 to 3.0) in preventing thromboembolism among 20,500 patients of atrial fibrillation [36].

## Dabigatran etexilate

Dabigatran etexilate is an orally active pro-drug which is rapidly converted to dabigatran by esterase-mediated hydrolysis in the plasma and the liver [37]. Dabigatran directly and reversibly inhibits both free and fibrin-bound thrombin, thereby interrupting the coagulation cascade. The oral bioavailability of this drug is approximately 6%-7%, which is much lower than that of the other three oral anticoagulants [1,4]. It has a half-life of 14 to 17 hours. Dabigatran is predominately (80%) excreted unchanged by the kidneys and its plasma levels are increased in patients with renal insufficiency [37,38]. It is contraindicated in patients with severe renal dysfunction (creatinine clearance <30 ml/min) [39].

Dabigatran is not metabolized by cytochrome P450 isoenzymes. Co-administration of dabigatran with atorvastatin (a substrate for CYP3A4 and P-glycoprotein) [40], digoxin (a P-glycoprotein substrate) [41] or diclofenac (a CYP2C9 substrate) [42] did not result in clinically relevant drug interactions. However, dabigatran etexilate (not the active dabigatran) acts as a substrate for Pglycoprotein. Therefore, drugs that inhibit P-glycoprotein, such as quinidine, verapamil and amiodarone, will increase the plasma levels of dabigatran [43]. Administration of dabigatran etexilate with food prolonged the time to peak plasma levels by approximately 2 hours without significantly affecting overall exposure [44].

### Orthopedic surgery

On the basis of the results of phase 2 studies demonstrating that dabigatran is effective for the prevention of venous thromboembolism (VTE), phase 3 studies for thromboprophylaxis after hip or knee arthroplasty were conducted. In the RENOVATE I trial [45], 3494 patients undergoing total hip replacement were randomized to treatment for 28 to 35 days with 220 mg or 150 mg dabigatran once daily initiated after surgery, or subcutaneous enoxaparin 40 mg once daily, initiated preoperatively. The primary efficacy outcome (total VTE or death) occurred in 6%, 8.6% and 6.7% of patients in the dabigatran 220 mg, 150 mg and enoxaparin groups, respectively. Risk differences were −0.7% (95% CI, -2.9 to 1 · 6%) and 1.9% (95% CI, −0 · 6 to 4 · 4%) for dabigatran 220 mg and 150 mg, respectively, compared with enoxaparin. Moreover, the incidence of major bleeding events did not differ significantly with either dose of dabigatran compared with enoxaparin (p = 0.44 for 220 mg, p = 0.60 for 150 mg). Similarly, in the REMODEL trial [46] enrolling 2076 patients undergoing total knee replacement, 6 to 10 days of treatment with either dose of dabigatran had efficacy similar to that of enoxaparin (enoxaparin 37.7%; 220 mg dabigatran, 36.4%; 150 mg dabigatran, 40.5%). Also, the incidence of major bleeding events did not differ significantly between the three groups (1.3% versus 1.5% and 1.3%, respectively).

The RENOVATE II trial [47] compared 28 to 35 days of treatment with dabigatran 220 mg once daily with subcutaneous enoxaparin 40 mg once daily for prevention of VTE in 2055 patients undergoing total hip arthroplasty. In this trial, the primary efficacy outcome occurred in 7.7% of the patients receiving dabigatran versus 8.8% of the patients receiving enoxaparin. Risk difference was −1.1% (95% CI, -3.8 to 1.6%); p < 0.0001 for the pre-specified non-inferiority margin. The rates of primary efficacy outcome observed in this trial were consistent with those observed in REVOVATE I [45]. Major bleeding events occurred in 1.4% of the patients in the dabigatran group and in 0.9% of the patients in the enoxaparin group (P = 0.40).

In the RE-MOBILIZE trial [48] of 1896 patients undergoing total knee arthroplasty, the North American enoxaparin regimen (30 mg BID postoperatively) was used. Treatment with either dose (220 mg or 150 mg) of dabigatran for 12 to 15 days was found to be statistically inferior to enoxaparin treatment for a similar duration (venous thromboembolism rates of 31% for 220 mg dabigatran [p = 0.02 vs. enoxaparin], 34% for 150 mg dabigatran [p < 0.001 vs. enoxaparin], and 25% with enoxaparin). This suggests that enoxaparin 30 mg twice daily is substantially more effective than enoxaparin 40 mg once daily for the prevention of VTE in patients undergoing knee arthroplasty. However, there were no significant differences in the incidence of major bleeding events (0.6%, 0.6%, and 1.4%, respectively).

### Atrial fibrillation

The Randomized Evaluation of Long-term Anticoagulant Therapy (RE-LY) trial [49] enrolled 18,113 patients with atrial fibrillation who were at risk for stroke. Two doses of dabigatran, 110 mg or 150 mg twice daily, administered in a blinded fashion, were compared with adjusted-dose warfarin, which was administered in an unblended fashion. The rate of primary outcome (systemic embolism or stroke) was 1.69% per year for warfarin, as compared with 1.11% per year for dabigatran at dose of 150 mg twice daily (relative risk, 0.66; 95% CI, 0.53 to 0.82; p < 0.001 for superiority) and 1.53% per year for dabigatran at a dose of 110 mg twice daily (relative risk with dabigatran, 0.91; 95% CI, 0.74 to 1.11; p < 0.001 for noninferiority.) Compared with warfarin (3.36% per year), however, the risk of major bleeding was 2.71% per year for dabigatran at a dose of 110 mg (p = 0.003), but similar (3.11% per year) for dabigatran at a dose of 150 mg (p = 0.31). Both doses of dabigatran were therefore better than warfarin.

### Acute coronary syndrome

In patients with recent ST-elevation or non ST-elevation MI, dabigatran was given in addition to dual antiplatelet therapy in a double-blind, placebo-controlled, dose-escalation phase 2 (RE-DEEM) trial [50]. A total of 1861 patients were randomized to twice daily treatment with dabigatran 50 mg (n = 369), 75 mg (n = 368), 110 mg (n = 406), 150 mg (n = 347), or placebo (n = 371). This trial showed that 3.8% of the patients in the placebo group had an MI, stroke or died compared with dabigatran at different doses; 4.6% in patients treated with 50 mg, 4.9% for 75 mg, 3% for 110 mg and 3.5% for those treated with 150 mg dabigatran.

Additionally, the incidence of major or clinically relevant minor bleeding events during the 6-month treatment period increased in a dose-dependent manner with dabigatran; the hazard ratio was 1.77 for 50 mg, 2.17 for 75 mg, 3.92 for 110 mg and 4.27 for 150 mg dabigatran compared with placebo.

The RE-LY trial [49] suggested an increased risk of MI with the use of dabigatran versus warfarin in patients with atrial fibrillation. In order to evaluate the risk of MI or ACS with dabigatran, a meta-analysis of seven trials of dabigatran was conducted [51]. Control agents in the studies included warfarin, enoxaparin and placebo administration. The results showed that the risk of MI or ACS was significantly higher in the 20,000 dabigatran patients than in the 10,514 control patients (1.19% vs. 0.79%, p = 0.03). The risk of MI or ACS was similar when using revised RE-LY trial results (95% CI, 1.00 to 1.61; p = 0.05).

### Symptomatic venous thromboembolism

In the RE-COVER 1 trial [52], patients with acute venous thromboembolism were randomized double-blind to six months of treatment with either dabigatran 150 mg twice daily or warfarin that was dose-adjusted to achieve an INR of 2.0 to 3.0 after initial treatment with a parenteral anticoagulant. Recurrent venous thromboembolism or related deaths occurred in 30 of the 1274 patients (2.4%) in the dabigatran group as compared with 27 of the 1265 patients (2.1%) in the warfarin group (95% CI, −0.8 to 1.5; p < 0.001 for the prespecified noninferiority margin). The hazard ratio with dabigatran was 1.10 (95% CI, 0.65 to 1.84). Major bleeding episodes in patients assigned to dabigatran (1.6%) were also at the same rate (1.9%) as for patients assigned to warfarin (hazard ratio with dabigatran, 0.82; 95% CI, 0.45 to 1.48).

## Conclusion

In this review, we have summarized the evidence from phase 3 clinical trials pertaining to the efficacy of apixaban, rivaroxaban, edoxaban and dabigatran in commonly encountered clinical conditions such as acute coronary syndrome, atrial fibrillation, symptomatic venous thromboembolism and orthopedic surgery. Our review suggests that the new oral anticoagulants represent a safe promising new class of drugs with potential to replace warfarin in the future. As these agents become more commonly used in clinical practice, it is imperative for the physicians to be aware of their pharmacological properties. Also, the future studies should attempt to identify effective reversal agents to be used in case of an adverse effect, such as a major bleed.

## Competing interests

The authors declared that they have no competing interest.

## Authors’ contributions

HR devised the manuscript, FT and HR did the literature search. FT, HR and TR wrote the manuscript. IBR edited the manuscript, HM and AH did the final revision. All the authors have read and approved the final version of the manuscript.
